# Response kinetics and depth of response after idecabtagene vicleucel in relapsed/refractory multiple myeloma

**DOI:** 10.1038/s41408-026-01582-z

**Published:** 2026-07-16

**Authors:** Utkarsh Goel, Catalin Dragomirescu, Roy Chedid, Diana Basali, Louis Williams, Sandra Mazzoni, Jason Valent, Shahzad Raza, Faiz Anwer, Jack Khouri

**Affiliations:** 1 Department of Internal Medicine, Cleveland Clinic, Cleveland, OH USA; 2 Department of Hematology and Medical Oncology, Taussig Cancer Center, Cleveland Clinic, Cleveland, OH USA; 3 Department of Medicine, University of Cincinnati, Cincinnati, OH USA

**Keywords:** Myeloma, Cancer immunotherapy

Dear Editor,

Chimeric Antigen Receptor T cell (CAR-T) therapy induces heterogenous depth of response and response kinetics in relapsed/refractory multiple myeloma (RRMM). E.g., in the KARMMA-1 trial, the median time to first response was 1 month (range 0.5–8.8), while the median time to complete response or better was also variable (median 2.8 months, range 1–11.8) [1]. This post-infusion disease behavior may not be fully captured by pre-infusion risk-stratification approaches alone [2–6]. While pre-infusion disease characteristics can be modified through clinical decision making, established interventions to modify post-infusion disease kinetics remain limited after CAR-T infusion, and subsequent disease trajectory is largely driven by the underlying tumor-CAR-T interaction. The implications of MM response kinetics early in newly diagnosed MM have been described, however the prognostic implications of post-CAR-T response and relapse kinetics remain incompletely defined [7, 8]. In the KARMMA-1 trial (median 6 prior lines of therapy, LOT), best response to idecabtagene vicleucel (ide-cel) was a significant predictor of progression-free survival (PFS) [1]. However, it is unclear whether depth of response remains a significant predictor of outcomes in patients with late-line MM (e.g., >8–10 prior LOT). In this study, we evaluated whether depth of response predicts outcomes even in heavily pre-treated MM and examined how post-infusion response/relapse kinetics influence subsequent survival.

We used data from the Center for International Blood and Marrow Transplant Research (CIBMTR) MM23-01a study, that reported outcomes after standard of care (SOC) ide-cel in the United States between May 2021 and June 2023 [9]. We studied the impact of depth of response after ide-cel and number of prior LOT on PFS and overall survival (OS) using the Kaplan–Meier method. We defined PFS as time until progression or death, and OS as time until death from any cause or last follow-up.

When studying time to best response (TBR) and duration of best response (time from first documented best response to PFS event, DBR), we calculated PFS and OS from the landmark time of first detectable best response (BR) to mitigate immortal time bias. We modeled TBR and DBR as continuous variables using Cox proportional hazards regression with restricted cubic splines to characterize their association with post-BR PFS and post-BR OS. To aid clinical interpretation, we explored a cutoff point corresponding to the earliest value at which the spline-estimated hazard ratio (HR) crossed 1, relative to the reference value. We then used these cutoffs for subsequent secondary analyses. Finally, we descriptively evaluated the impact of response/relapse kinetics on post-BR PFS and post-BR OS using univariable and multivariable Cox models including age, cytogenetic risk, receipt of prior BCMA-directed therapy, extramedullary plasmacytoma involvement as captured in the CIBMTR dataset, ECOG performance status, and best response to ide-cel.

A total of 821 patients were included with a median of 7 prior LOT (IQR 6–9), Table S1. Of the 821 patients, 808 had best response information available. The overall response rate (ORR) was 73%, 31% achieved a very good partial response (VGPR), and 25% achieved a complete response (CR), Fig. S1. The median PFS and OS from infusion were 8.75 months (95% CI: 7.8–10.7) and 15.6 months (95% CI: 13.8–17.8), respectively.

Best response after ide-cel was a significant predictor of PFS. The median PFS with a best response of PR was 7 months (95% CI: 6.4–9) compared to 11.8 months (95% CI: 10.7-NR) for VGPR, and NR (95% CI: 13.3-NR) for CR, *p* < 0.001. The median OS with the best response of PR was 13 months (95% CI: 10.8-NR) compared to NR (95% CI: 22-NR) for VGPR, and 18 months (95% CI: 16-NR) for those achieving a CR. Depth of response remained a significant predictor of PFS OS across all prior LOT groups (4–6 LOT, 7–9 LOT, and ≥10 LOT, Fig. 1). When analyzing LOT as a continuous variable, depth of response predicted PFS and OS in an incremental fashion across all prior LOT (Figs. S2 and S3). There was no significant interaction between best response and prior lines for PFS (*p* = 0.52) or OS (*p* = 0.61).

**Fig. 1:**
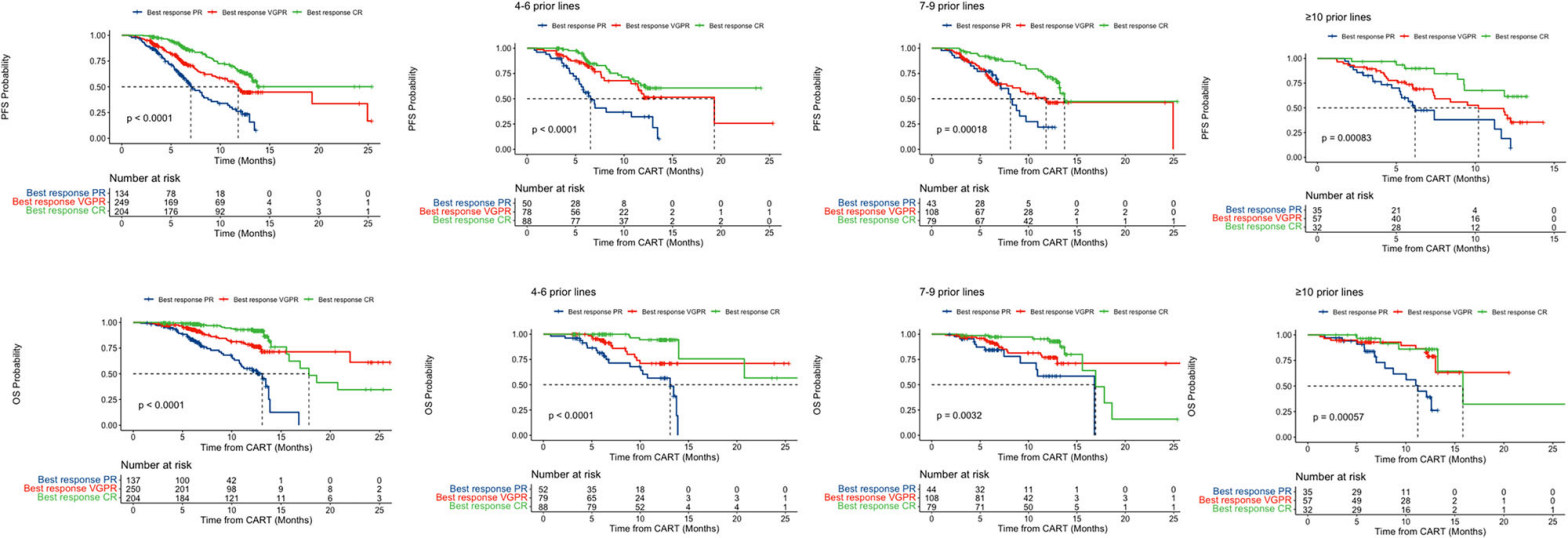
Impact of depth of response on PFS and OS based on number of prior lines of therapy. PFS indicates progression free survival, OS overall survival, LOT lines of therapy, PR partial response, VGPR very good partial response, CR complete response.

All subsequent analyses included patients who achieved at least a PR and had complete TBR and PFS information available (*n* = 503). Restricted cubic splines identified cutoffs of 3 months for TBR and 4 months for duration of best response (DBR, Figs. S4, S5, and S8).

Based on the TBR cutoff (3 months), there were no significant differences in the baseline characteristics in the two TBR cohorts, except that patients with a shorter TBR had a higher prevalence of extramedullary disease (30% vs 11%, *p* = 0.038, Table S3). TBR of >3 months was associated with a superior post-BR PFS as compared to TBR ≤ 3 months (10.2 months [95% CI: 8-NR] vs 6.6 months [95% CI: 5.9–8.9], 0.62 [95% CI: 0.45–0.84]) on univariable analysis, however, was not statistically significant when the cohort was stratified by best response (Fig. S10). On multivariable analysis, TBR was not independently associated with post-BR PFS (HR for >3 months 0.92 [95% CI: 0.59–1.43], Fig. S6). Similar trends were observed for post-BR OS (Figs. S7 and S10).

Based on TBR and DBR cutoffs, patients were classified into 4 groups: late response/late relapse (*n* = 96, 19%), late response/early relapse (*n* = 105, 21%), early response/late relapse (*n* = 156, 31%) and early response/early relapse (*n* = 146, 29%). Patients in the late response/late relapse category had a higher proportion of ISS stage I disease (57% vs 51–55% for the other groups, *p* = 0.033, Table S4). The late response/late relapse group had the best post-BR OS (median NR, 95% CI: 14.6-NR), while the early response/early relapse group had an inferior OS to the other groups (median 7.4 months, 95% CI: 5.6–10.7, *p* < 0.001, Fig. 2). Response/relapse kinetics were also significantly associated with overall best response. Patients achieving ≥VGPR or ≥CR as best response were more likely to have a late response/late relapse phenotype (Table S5). On univariable analysis, the HR for post-BR OS for early response/early relapse was 13.7 (95% CI: 6.1–30.6), late response/early relapse was 12.6 (95% CI: 5.2–30.7), and early response/late relapse was 2.1 (95% CI: 0.9–4.8) as compared to the late response/late relapse reference group. This trend was maintained on multivariable analysis, although these findings should be interpreted descriptively because relapse timing is inherently related to duration of response and subsequent survival (Fig. S9).

**Fig. 2:**
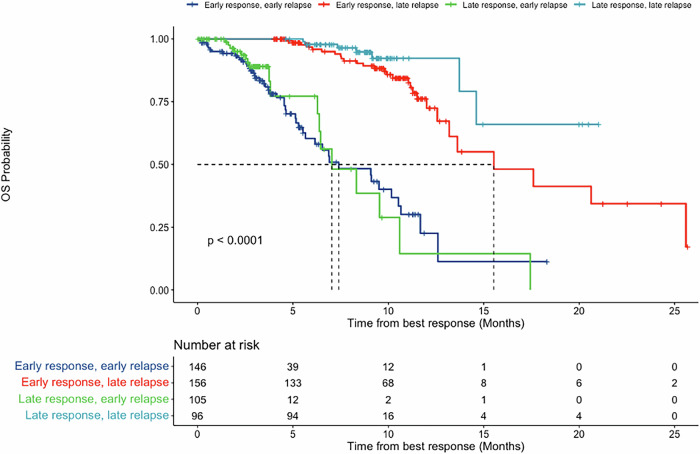
Impact of post-CAR T response and relapse kinetics on post-BR OS. CAR T indicates chimeric antigen receptor T-cell therapy, BR best response, OS overall survival.

In clinical practice, patients with late-line MM often derive meaningful benefit from RRMM therapies despite not achieving deep responses, raising the question of whether pursuing deep responses remains the appropriate therapeutic goal in heavily pretreated disease. While favorable outcomes despite suboptimal responses in this setting may partly reflect selection for more indolent disease biology, depth of response remained prognostically relevant even in a heavily pretreated population and across all prior LOT in our study. These observations raise the possibility that select patients with suboptimal responses to SOC CAR-T may benefit from future studies evaluating post-infusion maintenance strategies to achieve the deepest response possible. Because management after CAR-T infusion is largely observational, clinicians and patients often monitor the evolving response trajectory in anticipation of subsequent treatment decisions. Our findings suggest that the timing of the initial response alone may not independently predict subsequent outcomes and should be interpreted within the overall context of response depth and durability.

Several retrospective studies examining response kinetics in multiple myeloma have reported conflicting results. Studies by Yan et al. and Mellors et al. found that response timing influenced outcomes, with shorter TBR being associated with inferior survival [8, 10]. Similarly, Lahuerta et al. observed that patients with post-autologous stem cell transplantation response deepening had superior outcomes, suggesting slower response trajectories may reflect favorable disease biology [11]. In contrast, Tandon et al. reported that while achieving ≥VGPR predicted improved survival, the rapidity of response did not [7]. In our cohort, shorter TBR was associated with inferior outcomes on univariable analysis, representing one of the more notable observations of this study. One possible explanation is that rapid attainment of best response may identify patients with more aggressive or proliferative disease biology, in whom early cytoreduction does not translate into durable disease control. However, this association was attenuated in multivariable analysis, suggesting that the apparent prognostic effect of TBR may be partly mediated by its relationship with eventual response depth and durability.

Our study is prone to several limitations. First, the cohort included only patients who received SOC ide-cel and achieved at least a PR; therefore, the results may not be directly applicable to patients who do not demonstrate an initial response to CAR-T therapy. These non-responders likely represent a particularly high-risk population and warrant consideration of early salvage strategies. Second, as a secondary analysis of registry-based data, the study remains susceptible to residual confounding. Third, the relatively short follow-up period may limit the ability to capture late relapses, which could influence the thresholds used to define early versus late events. Fourth, because the response/relapse trajectory groups incorporate duration of best response, they are inherently related to PFS and should be interpreted as descriptive phenotypes of post-infusion disease behavior rather than as an independent prognostic model. Additionally, time to ORR and TBR were identical among patients with both variables available (Table S2), indicating that TBR did not distinguish initial response timing from subsequent response deepening in this registry-based dataset. This may reflect both registry-based response-date capture and the rapid early antimyeloma activity observed after CAR-T therapy, whereby initial response and best documented response may occur within a similar early post-infusion assessment window. Finally, because the cohort consisted exclusively of patients treated with ide-cel, the findings may not be directly generalizable to other CAR-T products that may exhibit different post-infusion kinetics.

In conclusion, post-CAR-T disease behavior in RRMM is better contextualized by response depth and durability than by TBR alone. In this registry-based cohort, TBR had limited independent prognostic value, while deeper and more durable responses were associated with superior outcomes. These readily available post-infusion response measures may help refine prognosis and inform future studies of risk-adapted post-CAR-T strategies.

## Supplementary information

Supplemental Material

## Data Availability

This dataset was collected by the Center for International Blood and Marrow Transplant Research (CIBMTR) which is supported primarily by the Public Health Service U24CA076518 from the National Cancer Institute; the National Heart, Lung, and Blood Institute; the National Institute of Allergy and Infectious Diseases; 75R60222C00011 from the Health Resources and Services Administration; N00014-24-1-2057 and N00014-25-1-2146 from the Office of Naval Research; NMDP (National Marrow Donor Program); and the Medical College of Wisconsin. All datasets analyzed for this study are publicly available at https://cibmtr.org/CIBMTR/Resources/Publicly-Available-Datasets.
