# Supplementary material for: Response kinetics and depth of response after idecabtagene vicleucel in relapsed/refractory multiple myeloma
Source: Blood Cancer J. 2026 Jul 16;16(1):116. doi: 10.1038/s41408-026-01582-z (PMC13377054; doi:10.1038/s41408-026-01582-z)
Supplement: Supplementary file 1 — Supplemental Material [file 41408_2026_1582_MOESM1_ESM.docx]

**Supplementary material**

**Contents**

Table S1- Baseline characteristics prior to ide-cel infusion

Table S2- CRS and ICANS within the first 100 days post-infusion, time to overall response and best response

Table S3- Baseline characteristics based on TBR cutoff.

Table S4- Baseline characteristics based on TBR and DBR cutoff.

Table S5- Depth of response distribution based on response kinetics.

Figure S1- Best response to ide-cel

Figure S2- Relation between prior lines of therapy and depth of response in predicting HR for PFS

Figure S3- Relation between prior lines of therapy and depth of response in predicting HR for OS

Figure S4- Effect of Time to best response on post-BR PFS.

Figure S5- Effect of Time to best response on post-BR OS.

Figure S6- Multivariable analysis for TBR in predicting post-BR PFS

Figure S7- Multivariable analysis for TBR in predicting post-BR OS

Figure S8- Effect of DBR on post-BR OS.

Figure S9- Multivariable analysis for response/ relapse kinetics in predicting post-BR OS.

Figure S10- Impact of Time to best response on post-BR PFS and OS.

**Table S1- Baseline characteristics prior to ide-cel infusion**

| **Characteristic** | **N = 821** |
| --- | --- |
| **Age at infusion, years** | 66 (59, 71) |
| **Sex** |  |
| Female | 334/820 (41%) |
| Male | 486/820 (59%) |
| Missing | 1 |
| **Race** |  |
| Black | 120/803 (15%) |
| Other | 31/803 (3.9%) |
| White | 652/803 (81%) |
| Missing | 18 |
| **Ethnicity** |  |
| Hispanic or Latino | 55/806 (6.8%) |
| Not Hispanic or Latino | 751/806 (93%) |
| Missing | 15 |
| **ECOG performance status at infusion** |  |
| ECOG ≥2 | 42/770 (5.5%) |
| ECOG 0-1 | 728/770 (95%) |
| Missing | 51 |
| **ISS stage at infusion** |  |
| Stage I | 210/420 (50%) |
| Stage II | 142/420 (34%) |
| Stage III | 68/420 (16%) |
| Missing | 401 |
| **Plasma cell leukemia** | 13/821 (1.6%) |
| **Extramedullary disease** | 85/488 (17%) |
| Missing | 333 |
| **Non-secretory myeloma** | 17/821 (2.1%) |
| **High BM PC % (>50%)** | 71/508 (14%) |
| Missing | 313 |
| **High-risk cytogenetics (FISH)** |  |
| High risk | 381/727 (52%) |
| Standard risk | 346/727 (48%) |
| Missing | 94 |
| **Number of prior lines of therapy** | 7.00 (6.00, 9.00) |
| Missing | 36 |
| **Prior autologous stem cell transplant** |  |
| No prior HCT | 113/805 (14%) |
| Prior allo-HCT | 9/805 (1.1%) |
| Prior auto and allo-HCT | 13/805 (1.6%) |
| Prior auto-HCT | 670/805 (83%) |
| Missing | 16 |
| **Prior BCMA-directed therapy** |  |
| Belantamab | 113/821 (14%) |
| CAR-T | 2/821 (0.2%) |
| More than one type of BCMA therapy | 3/821 (0.4%) |
| No | 700/821 (85%) |
| Teclistamab | 3/821 (0.4%) |
| **Triple-refractory disease** | 776/820 (95%) |
| **Penta-refractory disease** | 490/820 (60%) |
| **Bridging therapy** | 442/799 (55%) |
| **Lymphodepleting chemotherapy** |  |
| Bendamustine | 51/820 (6.2%) |
| Fludarabine + cyclophosphamide | 741/820 (90%) |
| Other | 28/820 (3.4%) |
| **Leukapheresis to infusion, days** | 46 (42, 50) |
| **Year of CAR-T infusion** |  |
| 2021 | 269/821 (33%) |
| 2022 | 454/821 (55%) |
| 2023 | 98/821 (12%) |

Values indicate median (IQR) for continuous variables. Percentages are calculated using non-missing values. Abbreviations: Ide-cel indicates idecabtagene vicleucel; ECOG, Eastern Cooperative Oncology Group; ISS, International Staging System; BM PC, Bone marrow plasma cells; HCT, hematopoietic stem cell transplantation; BCMA, B-cell maturation antigen; CAR-T, chimeric antigen receptor T-cell therapy.

**Table S2- CRS and ICANS within the first 100 days post-infusion, time to overall response and best response**

| **Characteristic** | **N = 821** |
| --- | --- |
| **CRS (within 100 days)** |  |
| Grade 1 | 470/813 (58%) |
| Grade 2 | 155/813 (19%) |
| Grade 3 | 9/813 (1.1%) |
| Grade 4 | 11/813 (1.4%) |
| Grade 5 | 4/813 (0.5%) |
| No CRS | 164/813 (20%) |
| Missing | 8 |
| **ICANS (within 100 days)** |  |
| Grade 1 | 99/771 (13%) |
| Grade 2 | 43/771 (5.6%) |
| Grade 3 | 26/771 (3.4%) |
| Grade 4 | 12/771 (1.6%) |
| Grade 5 | 1/771 (0.1%) |
| No ICANS | 590/771 (77%) |
| Missing | 50 |
|  |  |
| **Time to overall response, months (N=592)*** | 2.9 months (1.4-3.5) |
| **Time to best response, months (N=550)*** | 2.8 months (1.32-3.48) |

Values indicate median (IQR) for continuous variables. Percentages are calculated using non-missing values. Abbreviations: CRS indicates cytokine release syndrome; ICANS, immune effector cell-associated neurotoxicity syndrome.

**Among patients with both variables available, time to overall response and time to best response were identical. Differences in summary estimates between these variables reflect different denominators due to missingness rather than meaningful separation between the two timepoints.*

**Table S3- Baseline characteristics based on TBR cutoff.**

| **Variable** | **N** | **Overall**  N = 503 | **TBR ≤3 months**  N = 302 | **TBR >3 months**  N = 201 | **p-value** |
| --- | --- | --- | --- | --- | --- |
| **Age at infusion, years** | 503 | 66 (59-72) | 66 (59-72) | 66 (59-73) | 0.7 |
| **Sex** | 503 |  |  |  | >0.9 |
| Female |  | 205 / 503 (41%) | 123 / 302 (41%) | 82 / 201 (41%) |  |
| Male |  | 298 / 503 (59%) | 179 / 302 (59%) | 119 / 201 (59%) |  |
| **Race** | 494 |  |  |  | 0.2 |
| Black |  | 68 / 494 (14%) | 34 / 296 (11%) | 34 / 198 (17%) |  |
| Other |  | 17 / 494 (3.4%) | 9 / 296 (3.0%) | 8 / 198 (4.0%) |  |
| White |  | 409 / 494 (83%) | 253 / 296 (85%) | 156 / 198 (79%) |  |
| **Ethnicity** | 497 |  |  |  | >0.9 |
| Hispanic or Latino |  | 22 / 497 (4.4%) | 13 / 298 (4.4%) | 9 / 199 (4.5%) |  |
| Not Hispanic or Latino |  | 475 / 497 (96%) | 285 / 298 (96%) | 190 / 199 (95%) |  |
| **ECOG performance status at infusion** | 468 |  |  |  | 0.2 |
| ECOG ≥2 |  | 21 / 468 (4.5%) | 15 / 275 (5.5%) | 6 / 193 (3.1%) |  |
| ECOG 0-1 |  | 447 / 468 (96%) | 260 / 275 (95%) | 187 / 193 (97%) |  |
| **ISS stage at infusion** | 255 |  |  |  | 0.2 |
| Stage I |  | 138 / 255 (54%) | 77 / 145 (53%) | 61 / 110 (55%) |  |
| Stage II |  | 80 / 255 (31%) | 42 / 145 (29%) | 38 / 110 (35%) |  |
| Stage III |  | 37 / 255 (15%) | 26 / 145 (18%) | 11 / 110 (10%) |  |
| **Plasma cell leukemia** | 503 | 7 / 503 (1.4%) | 6 / 302 (2.0%) | 1 / 201 (0.5%) | 0.3 |
| **Extramedullary disease** | 282 | **46 / 282 (16%)** | **32 / 157 (20%)** | **14 / 125 (11%)** | **0.038** |
| **Non-secretory myeloma** | 503 | 6 / 503 (1.2%) | 5 / 302 (1.7%) | 1 / 201 (0.5%) | 0.4 |
| **High BM PC % (>50%)** | 304 | 34 / 304 (11%) | 19 / 173 (11%) | 15 / 131 (11%) | 0.9 |
| **High-risk cytogenetics (FISH)** | 452 |  |  |  | >0.9 |
| High risk |  | 233 / 452 (52%) | 141 / 274 (51%) | 92 / 178 (52%) |  |
| Standard risk |  | 219 / 452 (48%) | 133 / 274 (49%) | 86 / 178 (48%) |  |
| **Number of prior lines of therapy** | 488 | 7.00 (6.00-9.00) | 7.00 (6.00-9.50) | 7.00 (6.00-9.00) | 0.050 |
| **Prior autologous stem cell transplant** | 496 |  |  |  | 0.8 |
| No prior HCT |  | 61 / 496 (12%) | 39 / 296 (13%) | 22 / 200 (11%) |  |
| Prior allo-HCT |  | 4 / 496 (0.8%) | 3 / 296 (1.0%) | 1 / 200 (0.5%) |  |
| Prior auto and allo-HCT |  | 6 / 496 (1.2%) | 3 / 296 (1.0%) | 3 / 200 (1.5%) |  |
| Prior auto-HCT |  | 425 / 496 (86%) | 251 / 296 (85%) | 174 / 200 (87%) |  |
| **Prior BCMA-directed therapy** | 503 |  |  |  | 0.5 |
| Belantamab |  | 59 / 503 (12%) | 39 / 302 (13%) | 20 / 201 (10.0%) |  |
| CAR-T |  | 2 / 503 (0.4%) | 1 / 302 (0.3%) | 1 / 201 (0.5%) |  |
| More than one type of BCMA therapy |  | 1 / 503 (0.2%) | 0 / 302 (0%) | 1 / 201 (0.5%) |  |
| No |  | 440 / 503 (87%) | 261 / 302 (86%) | 179 / 201 (89%) |  |
| Teclistamab |  | 1 / 503 (0.2%) | 1 / 302 (0.3%) | 0 / 201 (0%) |  |
| **Triple-refractory disease** | 502 | 477 / 502 (95%) | 288 / 302 (95%) | 189 / 200 (95%) | 0.7 |
| **Penta-refractory disease** | 502 | 302 / 502 (60%) | 188 / 302 (62%) | 114 / 200 (57%) | 0.2 |
| **Bridging therapy** | 490 | 270 / 490 (55%) | 161 / 293 (55%) | 109 / 197 (55%) | >0.9 |
| **Lymphodepleting chemotherapy** | 503 |  |  |  | 0.091 |
| Bendamustine |  | 16 / 503 (3.2%) | 13 / 302 (4.3%) | 3 / 201 (1.5%) |  |
| Fludarabine + cyclophosphamide |  | 475 / 503 (94%) | 284 / 302 (94%) | 191 / 201 (95%) |  |
| Other |  | 12 / 503 (2.4%) | 5 / 302 (1.7%) | 7 / 201 (3.5%) |  |
| **Leukapheresis to infusion, days** | 503 | 46 (42-50) | 45 (41-49) | 47 (42-51) | 0.027 |
| **Year of CAR-T infusion** | 503 |  |  |  | 0.003 |
| 2021 |  | 181 / 503 (36%) | 97 / 302 (32%) | 84 / 201 (42%) |  |
| 2022 |  | 271 / 503 (54%) | 164 / 302 (54%) | 107 / 201 (53%) |  |
| 2023 |  | 51 / 503 (10%) | 41 / 302 (14%) | 10 / 201 (5.0%) |  |

Values indicate median (IQR) for continuous variables. Percentages are calculated using non-missing values. Abbreviations: TBR indicates time to best response; ECOG, Eastern Cooperative Oncology Group; ISS, International Staging System; BM PC, Bone marrow plasma cells; HCT, hematopoietic stem cell transplantation; BCMA, B-cell maturation antigen; CAR-T, chimeric antigen receptor T-cell therapy. We used the analysis of variance (ANOVA) or the Kruskal-Wallis test to compare continuous variables, and the chi-square or Fischer’s exact test to compare categorical variables, as appropriate.

**Table S4- Baseline characteristics based on TBR and DBR cutoff.**

| **Variable** | **N** | **Overall**  N = 503 | **Late response / Late relapse**  N = 96 | **Late response / Early relapse**  N = 105 | **Early response / Late relapse**  N = 156 | **Early response / Early relapse**  N = 146 | **p-value** |
| --- | --- | --- | --- | --- | --- | --- | --- |
| **Age at infusion, years** | 503 | 66 (59-72) | 66 (59-73) | 67 (60-73) | 65 (59-71) | 67 (60-72) | 0.8 |
| **Sex** | 503 |  |  |  |  |  | 0.4 |
| Female |  | 205 / 503 (41%) | 34 / 96 (35%) | 48 / 105 (46%) | 60 / 156 (38%) | 63 / 146 (43%) |  |
| Male |  | 298 / 503 (59%) | 62 / 96 (65%) | 57 / 105 (54%) | 96 / 156 (62%) | 83 / 146 (57%) |  |
| **Race** | 494 |  |  |  |  |  | 0.2 |
| Black |  | 68 / 494 (14%) | 13 / 94 (14%) | 21 / 104 (20%) | 15 / 155 (9.7%) | 19 / 141 (13%) |  |
| Other |  | 17 / 494 (3.4%) | 3 / 94 (3.2%) | 5 / 104 (4.8%) | 7 / 155 (4.5%) | 2 / 141 (1.4%) |  |
| White |  | 409 / 494 (83%) | 78 / 94 (83%) | 78 / 104 (75%) | 133 / 155 (86%) | 120 / 141 (85%) |  |
| **Ethnicity** | 497 |  |  |  |  |  | 0.2 |
| Hispanic or Latino |  | 22 / 497 (4.4%) | 7 / 96 (7.3%) | 2 / 103 (1.9%) | 9 / 155 (5.8%) | 4 / 143 (2.8%) |  |
| Not Hispanic or Latino |  | 475 / 497 (96%) | 89 / 96 (93%) | 101 / 103 (98%) | 146 / 155 (94%) | 139 / 143 (97%) |  |
| **ECOG performance status at infusion** | 468 |  |  |  |  |  | 0.034 |
| ECOG ≥2 |  | 21 / 468 (4.5%) | 2 / 91 (2.2%) | 4 / 102 (3.9%) | 3 / 144 (2.1%) | 12 / 131 (9.2%) |  |
| ECOG 0-1 |  | 447 / 468 (96%) | 89 / 91 (98%) | 98 / 102 (96%) | 141 / 144 (98%) | 119 / 131 (91%) |  |
| **ISS stage at infusion** | 255 |  |  |  |  |  | **0.033** |
| Stage I |  | 138 / 255 (54%) | 26 / 46 (57%) | 35 / 64 (55%) | 38 / 74 (51%) | 39 / 71 (55%) |  |
| Stage II |  | 80 / 255 (31%) | 18 / 46 (39%) | 20 / 64 (31%) | 17 / 74 (23%) | 25 / 71 (35%) |  |
| Stage III |  | 37 / 255 (15%) | 2 / 46 (4.3%) | 9 / 64 (14%) | 19 / 74 (26%) | 7 / 71 (9.9%) |  |
| **Plasma cell leukemia** | 503 | 7 / 503 (1.4%) | 0 / 96 (0%) | 1 / 105 (1.0%) | 3 / 156 (1.9%) | 3 / 146 (2.1%) | 0.6 |
| **Extramedullary disease** | 282 | 46 / 282 (16%) | 6 / 53 (11%) | 8 / 72 (11%) | 17 / 82 (21%) | 15 / 75 (20%) | 0.2 |
| **Non-secretory myeloma** | 503 | 6 / 503 (1.2%) | 1 / 96 (1.0%) | 0 / 105 (0%) | 4 / 156 (2.6%) | 1 / 146 (0.7%) | 0.4 |
| **High BM PC % (>50%)** | 304 | 34 / 304 (11%) | 5 / 58 (8.6%) | 10 / 73 (14%) | 12 / 92 (13%) | 7 / 81 (8.6%) | 0.6 |
| **High-risk cytogenetics (FISH)** | 452 |  |  |  |  |  | 0.3 |
| High risk |  | 233 / 452 (52%) | 40 / 85 (47%) | 52 / 93 (56%) | 67 / 143 (47%) | 74 / 131 (56%) |  |
| Standard risk |  | 219 / 452 (48%) | 45 / 85 (53%) | 41 / 93 (44%) | 76 / 143 (53%) | 57 / 131 (44%) |  |
| **Number of prior lines of therapy** | 488 | 7.00 (6.00-9.00) | 7.00 (6.00-9.00) | 7.00 (6.00-8.00) | 7.00 (6.00-10.00) | 7.00 (6.00-9.00) | 0.2 |
| **Prior autologous stem cell transplant** | 496 |  |  |  |  |  |  |
| No prior HCT |  | 61 / 496 (12%) | 9 / 95 (9.5%) | 13 / 105 (12%) | 15 / 153 (9.8%) | 24 / 143 (17%) |  |
| Prior allo-HCT |  | 4 / 496 (0.8%) | 1 / 95 (1.1%) | 0 / 105 (0%) | 2 / 153 (1.3%) | 1 / 143 (0.7%) |  |
| Prior auto and allo-HCT |  | 6 / 496 (1.2%) | 2 / 95 (2.1%) | 1 / 105 (1.0%) | 3 / 153 (2.0%) | 0 / 143 (0%) |  |
| Prior auto-HCT |  | 425 / 496 (86%) | 83 / 95 (87%) | 91 / 105 (87%) | 133 / 153 (87%) | 118 / 143 (83%) |  |
| **Prior BCMA-directed therapy** | 503 |  |  |  |  |  | 0.3 |
| Belantamab |  | 59 / 503 (12%) | 6 / 96 (6.3%) | 14 / 105 (13%) | 18 / 156 (12%) | 21 / 146 (14%) |  |
| CAR-T |  | 2 / 503 (0.4%) | 1 / 96 (1.0%) | 0 / 105 (0%) | 1 / 156 (0.6%) | 0 / 146 (0%) |  |
| More than one type of BCMA therapy |  | 1 / 503 (0.2%) | 0 / 96 (0%) | 1 / 105 (1.0%) | 0 / 156 (0%) | 0 / 146 (0%) |  |
| No |  | 440 / 503 (87%) | 89 / 96 (93%) | 90 / 105 (86%) | 137 / 156 (88%) | 124 / 146 (85%) |  |
| Teclistimab |  | 1 / 503 (0.2%) | 0 / 96 (0%) | 0 / 105 (0%) | 0 / 156 (0%) | 1 / 146 (0.7%) |  |
| **Triple-refractory disease** | 502 | 477 / 502 (95%) | 93 / 95 (98%) | 96 / 105 (91%) | 147 / 156 (94%) | 141 / 146 (97%) | 0.2 |
| **Penta-refractory disease** | 502 | 302 / 502 (60%) | 52 / 95 (55%) | 62 / 105 (59%) | 101 / 156 (65%) | 87 / 146 (60%) | 0.5 |
| **Bridging therapy** | 490 | 270 / 490 (55%) | 51 / 95 (54%) | 58 / 102 (57%) | 87 / 152 (57%) | 74 / 141 (52%) | 0.8 |
| **Lymphodepleting chemotherapy** | 503 |  |  |  |  |  | <0.001 |
| Bendamustine |  | 16 / 503 (3.2%) | 0 / 96 (0%) | 3 / 105 (2.9%) | 3 / 156 (1.9%) | 10 / 146 (6.8%) |  |
| Fludarabine + cyclophosphamide |  | 475 / 503 (94%) | 95 / 96 (99%) | 96 / 105 (91%) | 153 / 156 (98%) | 131 / 146 (90%) |  |
| Other |  | 12 / 503 (2.4%) | 1 / 96 (1.0%) | 6 / 105 (5.7%) | 0 / 156 (0%) | 5 / 146 (3.4%) |  |
| **Leukapheresis to infusion, days** | 503 | 46 (42-50) | 47 (42-50) | 47 (42-52) | 46 (42-49) | 45 (41-50) | 0.2 |
| **Year of CAR-T infusion** | 503 |  |  |  |  |  | <0.001 |
| 2021 |  | 181 / 503 (36%) | 58 / 96 (60%) | 26 / 105 (25%) | 69 / 156 (44%) | 28 / 146 (19%) |  |
| 2022 |  | 271 / 503 (54%) | 38 / 96 (40%) | 69 / 105 (66%) | 82 / 156 (53%) | 82 / 146 (56%) |  |
| 2023 |  | 51 / 503 (10%) | 0 / 96 (0%) | 10 / 105 (9.5%) | 5 / 156 (3.2%) | 36 / 146 (25%) |  |

Abbreviations: TBR indicates time to best response; DBR, duration of best response; ECOG, Eastern Cooperative Oncology Group; ISS, International Staging System; BM PC, bone marrow plasma cells; FISH, fluorescent in situ hybridization; HCT, hematopoietic stem cell transplantation; BCMA, B-cell maturation antigen; CAR-T, chimeric antigen receptor T-cell therapy. We used the analysis of variance (ANOVA) or the Kruskal-Wallis test to compare continuous variables, and the chi-square or Fischer’s exact test to compare categorical variables, as appropriate.

**Table S5- Depth of response distribution based on response kinetics**

| **Variable** | **N** | **Overall**  N = 503 | **Late response / Late relapse**  N = 96 | **Late response / Early relapse**  N = 105 | **Early response / Late relapse**  N = 156 | **Early response / Early relapse**  N = 146 | **p-value** |
| --- | --- | --- | --- | --- | --- | --- | --- |
| **Best response** | 503 |  |  |  |  |  | <0.001 |
| PR |  | 124 / 503 (25%) | 13 / 96 (14%) | 22 / 105 (21%) | 36 / 156 (23%) | 53 / 146 (36%) |  |
| VGPR |  | 212 / 503 (42%) | 35 / 96 (36%) | 40 / 105 (38%) | 76 / 156 (49%) | 61 / 146 (42%) |  |
| CR |  | 167 / 503 (33%) | 48 / 96 (50%) | 43 / 105 (41%) | 44 / 156 (28%) | 32 / 146 (22%) |  |
| **≥VGPR** | 503 | 379 / 503 (75%) | 83 / 96 (86%) | 83 / 105 (79%) | 120 / 156 (77%) | 93 / 146 (64%) | <0.001 |
| **≥CR** | 503 | 167 / 503 (33%) | 48 / 96 (50%) | 43 / 105 (41%) | 44 / 156 (28%) | 32 / 146 (22%) | <0.001 |

Abbreviations: PR indicates partial response; VGPR, very good partial response; CR, complete response.

**Figure S1- Best response to ide-cel**


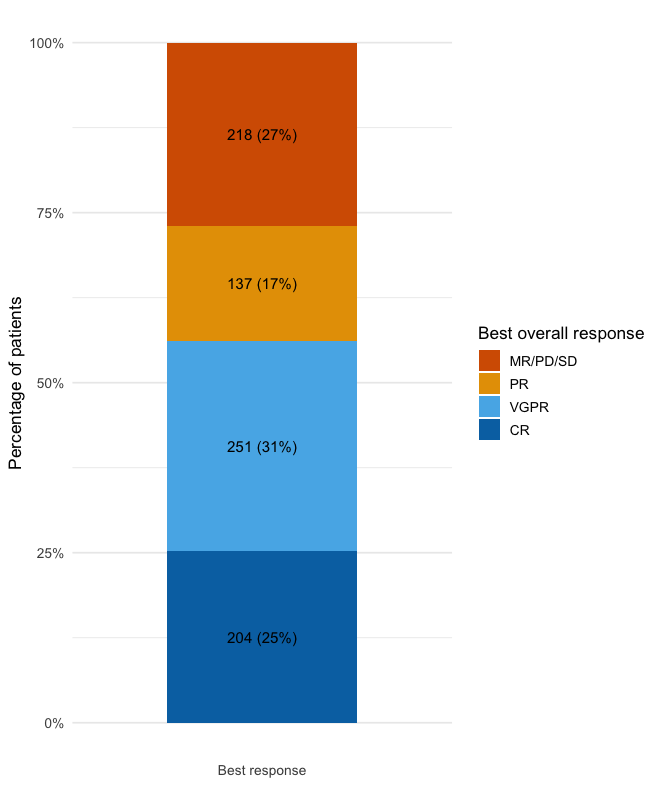


Abbreviations: Ide-cel indicates idecabtagene vicleucel; MR, minimal response; PD, progressive disease; SD, stable disease; PR, partial response; VGPR, very good partial response; CR, complete response.

**Figure S2 - Relation between prior lines of therapy and depth of response in predicting HR for PFS**

**
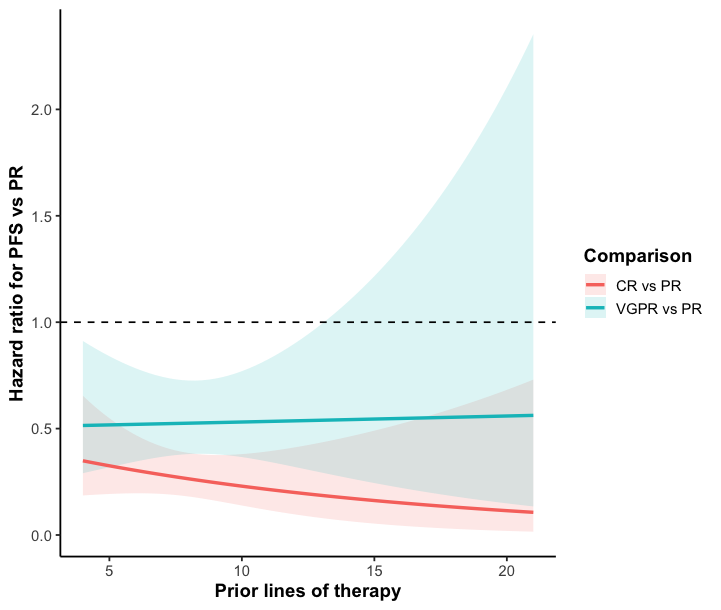
**

Abbreviations: PFS indicates progression free survival; HR, hazard ratio, PR, partial response; VGPR, very good partial response; CR, complete response.

**Figure S3- Relation between prior lines of therapy and depth of response in predicting HR for OS**

**
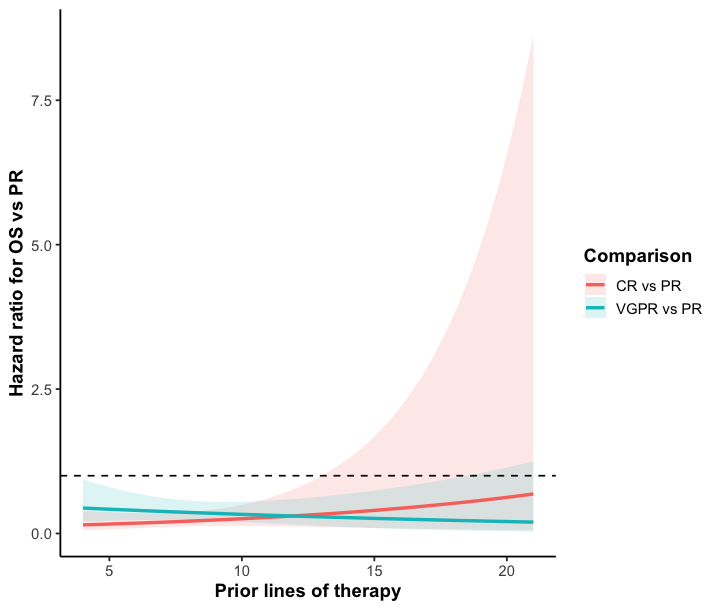
**

Abbreviations: OS indicates overall survival; HR, hazard ratio; PR, partial response; VGPR, very good partial response; CR, completer response.

**Figure S4- Effect of Time to best response on post-BR PFS.** A data-driven cutoff was defined as the earliest time point at which the spline-estimated hazard ratio crossed 1 relative to the reference value; shaded bands represent 95% confidence intervals. Spline analysis revealed an approximately linear association between time to best response and progression-free survival. The estimated hazard ratio crossed 1 at approximately 2.8 months; this value was rounded to 3 months for clinical interpretability.

**
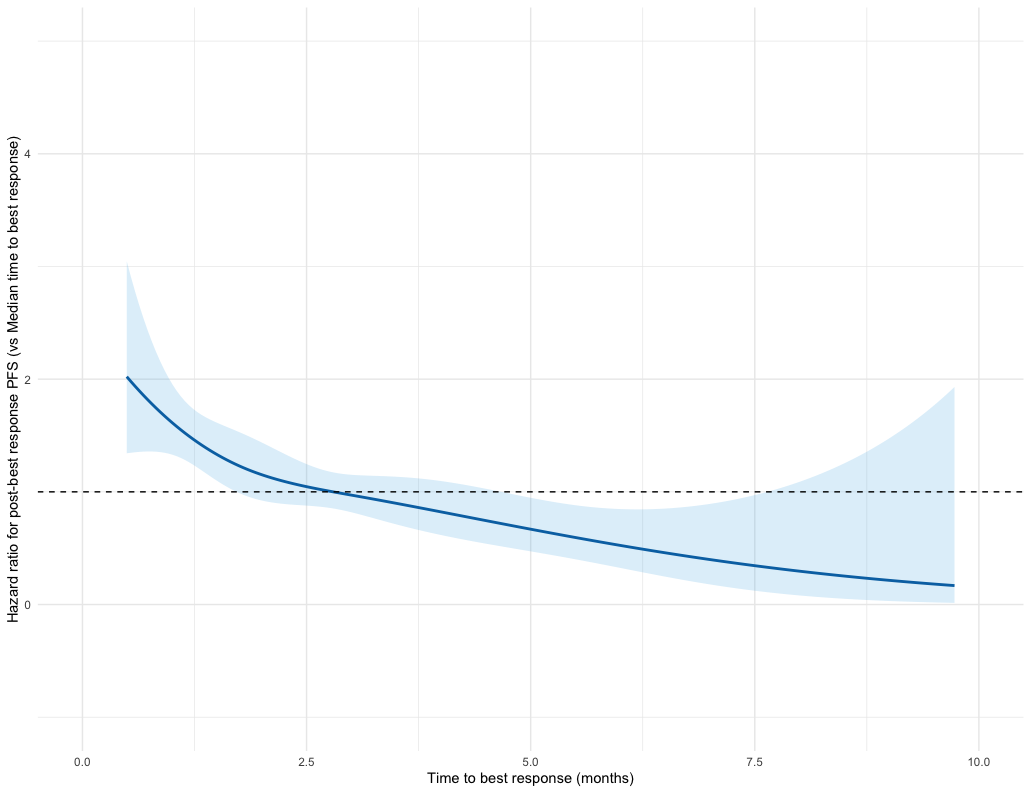
**

Abbreviations: BR indicates best response; PFS, progression free survival.

**Figure S5- Effect of Time to best response on post-BR OS.**

**
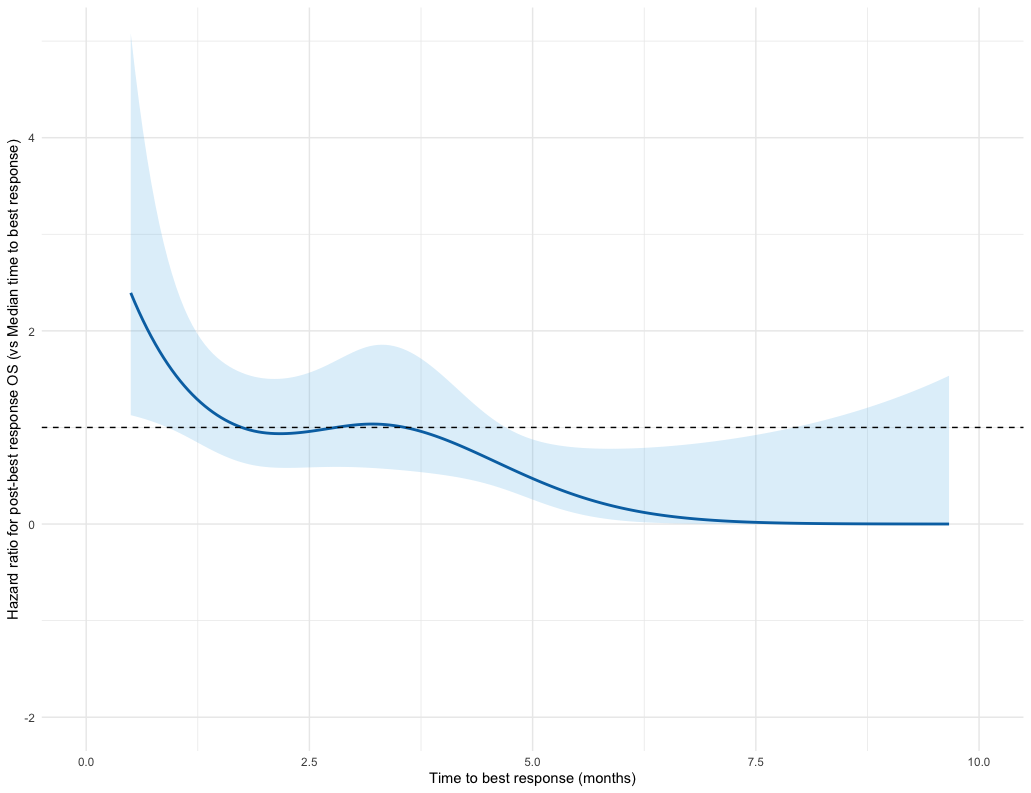
**

Abbreviations: BR indicates best response; OS, overall survival.

**Figure S6- Multivariable analysis for TBR in predicting post-BR PFS**

**
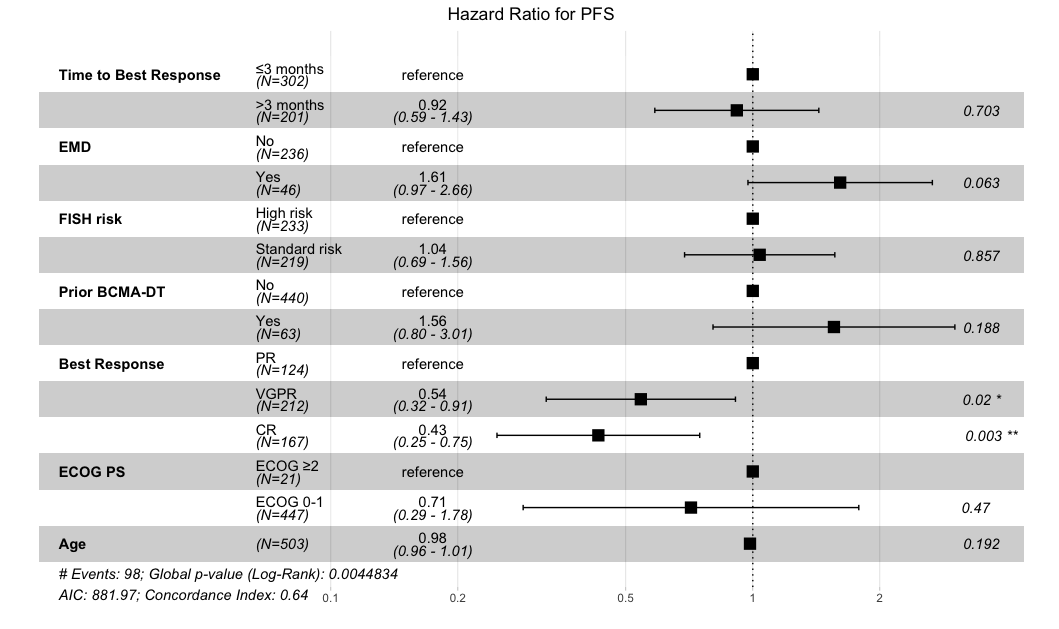
**

Abbreviations: TBR indicates time to best response; PFS, progression free survival; EMD, extramedullary disease; FISH, fluorescence in-situ hybridization; BCMA-DT, B-cell maturation antigen directed therapy; ECOG PS, Eastern Cooperative Oncology Group Performance Status.

**Figure S7- Multivariable analysis for TBR in predicting post-BR OS**

**
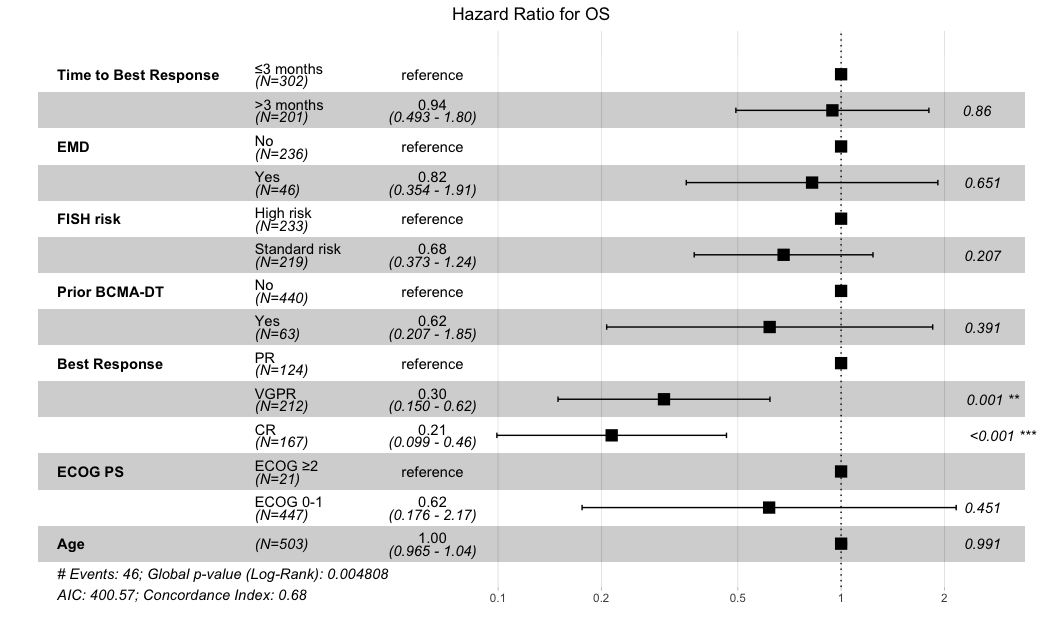
**

Abbreviations: TBR indicates time to best response; OS, overall survival; EMD, extramedullary disease; FISH, fluorescence in-situ hybridization; BCMA-DT, B-cell maturation antigen directed therapy; ECOG PS, Eastern Cooperative Oncology Group Performance Status.

**Figure S8- Effect of DBR on post-BR OS.** A data-driven cutoff was defined as the earliest time point at which the spline-estimated hazard ratio crossed 1 relative to the reference value; shaded bands represent 95% confidence intervals. Spline analysis revealed a nonlinear association between duration of best response and overall survival. The estimated hazard ratio crossed 1 at approximately 4.1 months; this value was rounded to 4 months for clinical interpretability.

**
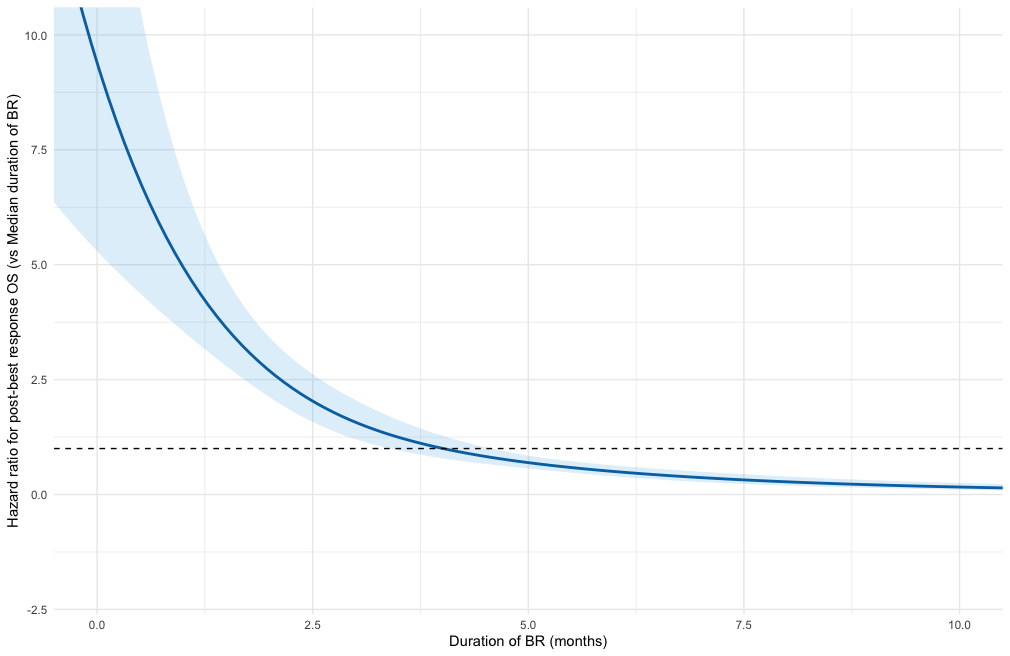
**

Abbreviations: DBR indicates duration of best response; OS, overall survival; BR, best response.

**
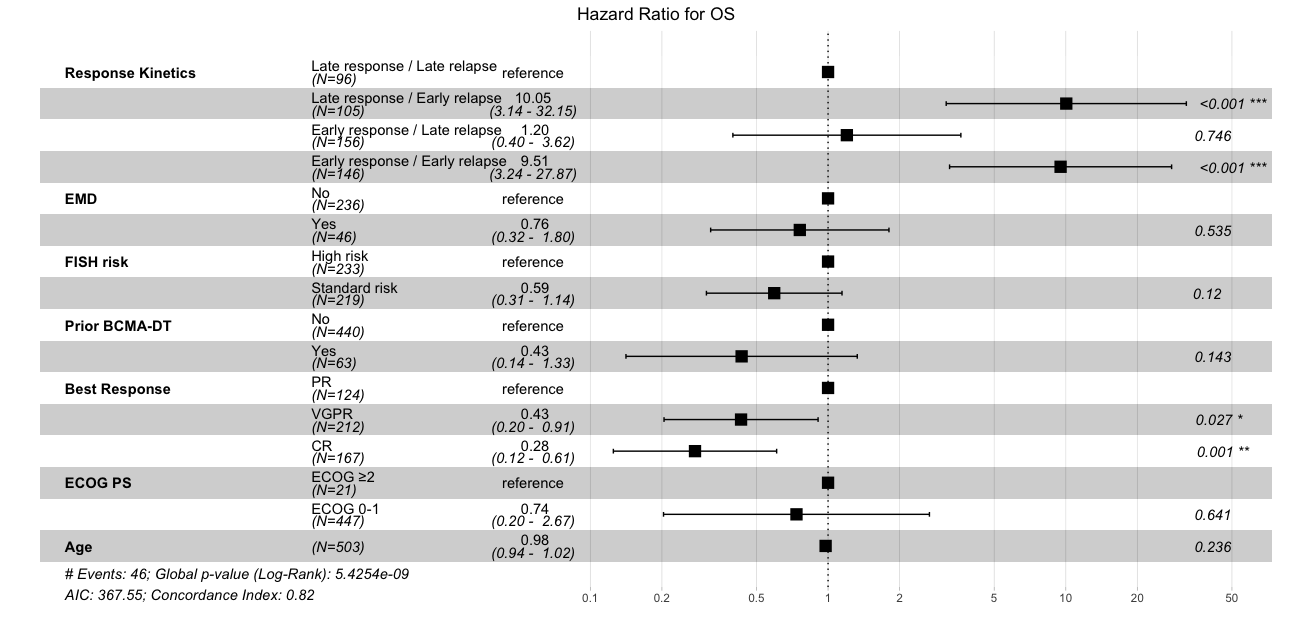
Figure S9- Multivariable analysis for response/ relapse kinetics in predicting post-BR OS.**

Abbreviations: BR indicates best response; OS, overall survival; EMD, extramedullary disease; FISH, fluorescence in-situ hybridization; BCMA-DT, B-cell maturation antigen directed therapy; ECOG PS, Eastern Cooperative Oncology Group Performance Status.

**
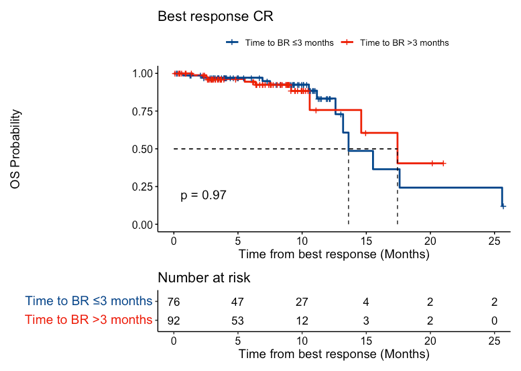

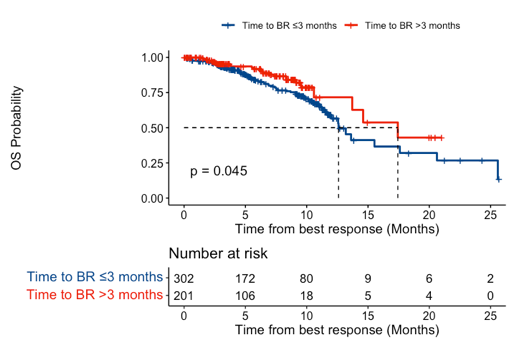

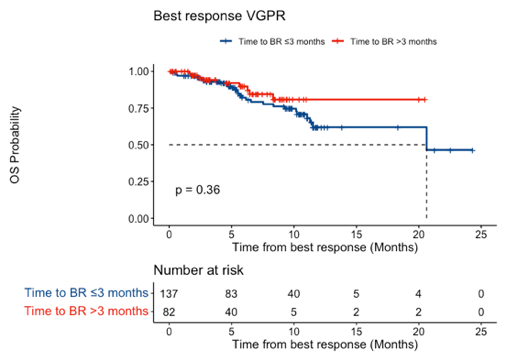

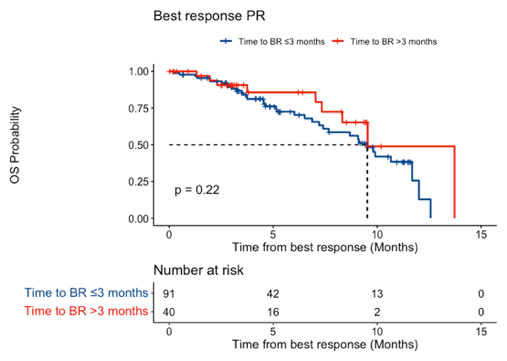

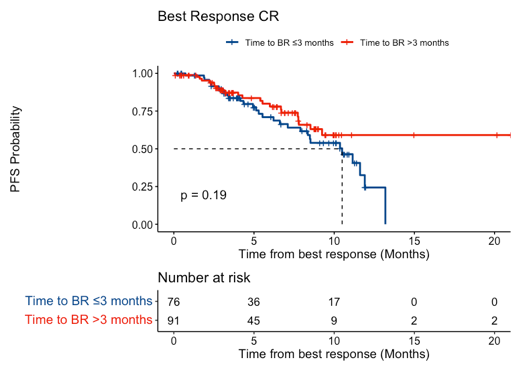

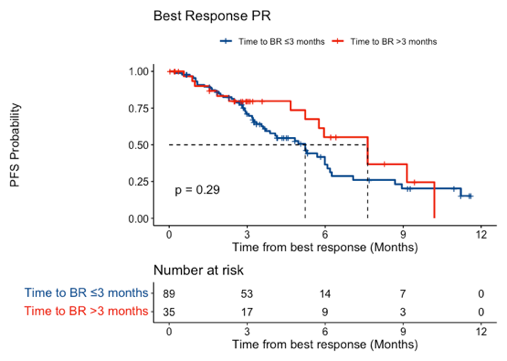

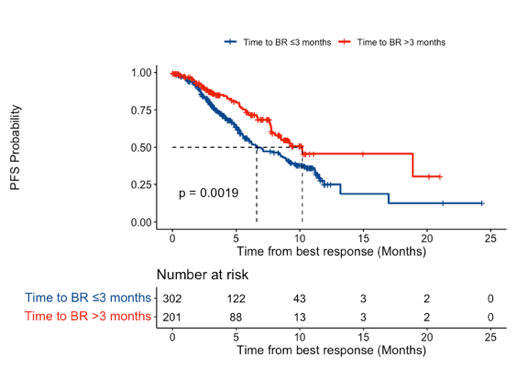
Figure S10- Impact of Time to best response on post-BR PFS and OS.**

**
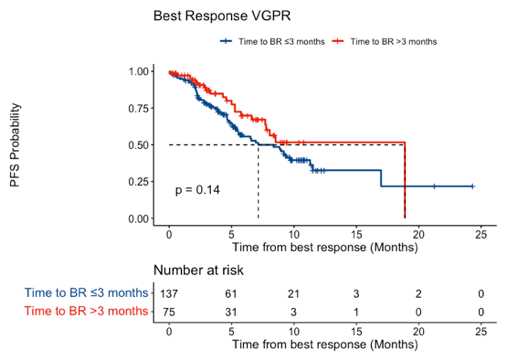
**

Abbreviations: BR indicates best response; PFS, progression free survival; OS, overall survival; PR, partial response; VGPR, very good partial response; CR, complete response.
